# Hejie Zhitong prescription promotes sleep and inhibits nociceptive transmission-associated neurotransmitter activity in a rodent migraine model

**DOI:** 10.1186/s13020-020-00386-y

**Published:** 2020-09-29

**Authors:** Xinna Wang, Hongfei Zhao, Liming Liu, Ping Niu, Chao Zhai, Jinjian Li, Qiaoli Xu, Dexi Zhao

**Affiliations:** 1 Changchun University of Traditional Chinese Medicine, Changchun, Jilin, 130117 China; 2 Administration of Traditional Chinese Medicine of Jilin Province, Changchun, Jilin, 130051 China

**Keywords:** Hejie Zhitong prescription, Sleep, Nociceptive transmission-associated neurotransmitter, Migraine

## Abstract

**Background:**

Migraine is painful disease in which neurotransmitters related to pain transmission play an important role. Hejie Zhitong prescription (HJZT) has been used in the clinic as an effective prescription for the treatment of migraine for many years. Our team aimed to further explore its antimigraine mechanism based on previous research results and to explore the inhibitory effect of HJZT on the transmission of pain related to nitroglycerine (NTG)-induced migraine as well as the synergistic effect of HJZT with pentobarbital sodium on promoting sleep.

**Methods:**

Sixty mice were randomly assigned to groups and received the corresponding interventions. Sleep latency and sleep time were recorded to calculate the incidence of sleep. Forty-eight Wistar rats were randomly assigned and administered an intervention corresponding to their group. Calcitonin gene-related peptide (CGRP), serotonin (5-HT), substance P (SP), and cholecystokinin (CCK) levels were measured using ELISAs. Levels of the cannabinoid receptor type 1 (CB1R) and cyclooxygenase-2 (COX-2) protein were assessed using immunohistochemistry. The expression of the CGRP and CCK mRNAs in the midbrain and trigeminal ganglion (TG) were measured using real-time quantitative PCR.

**Results:**

HJZT promoted the occurrence of sleep in mice. HJZT downregulated COX-2 expression in the midbrain and TG of rats but upregulated the expression of the CB1R, and decreased the plasma level of the CGRP protein and expression of its mRNA in the midbrain and TG. It also downregulated the expression of the CCK mRNA in the midbrain and TG. The high-dose HJZT treatment increased plasma 5-HT levels, but did not induce changes in the plasma levels of the SP or CCK protein.

**Conclusions:**

HJZT exerts a synergistic effect with pentobarbital sodium on promoting sleep. As for anti-migraine, HJZT can inhibits the expression of nociceptive transmission-associated neurotransmitters, including 5-HT, CGRP and CCK, which may be related to its upregulation of CB1R and downregulation of COX-2.

## Background

Migraine is a common clinical chronic neurovascular disease with paroxysmal and recurrent characters. It reduces patients’ quality of life and increases the economic burden [1].As the sixth most disabling disease worldwide, it has substantially affected humans for centuries [2, 3].The acute treatment of migraine mainly consists of the application of nonspecific analgesics, among which triptans have shown the best evidence of efficacy [4].Prophylactic treatment may reduce the frequency of pain, improve the quality of life of patients and prevent progression of the disease to chronic migraine [5],with which insomnia commonly co-occurs [6].As the study of migraine has progressively intensified, the hypotheses of its pathological mechanism have developed from a purely vascular to a neurovascular hypothesis, but the site of initial activation of the migraine process is uncertain. The hypothalamus and midbrain tegmentum may be involved [7], and pathological anatomical changes in areas such as the gray matter surrounding the midbrain sulcus have also been observed in migraineurs [8].Thus, researchers have proposed that areas such as the hypothalamus and midbrain may trigger and sustain the process of migraine attacks [9]. Recently, an increased emphasis has been placed on the role of the trigeminovascular system in connecting peripheral events with central consequences; specifically, trigeminal ganglion (TG) neurons provide the connection between the periphery by expressing the neuropeptide calcitonin gene-related peptide (CGRP) at high levels [10].

Herbal therapy has been used for thousands of years and may provide avenues for exploring therapeutic approaches for migraine. The results of one meta-analysis (Traditional Chinese Patent Medicine for Prophylactic Treatment of Migraine: A Meta-Analysis of Randomized, Double-Blind, Placebo-Controlled Trials) showed that a Chinese medicine treatment is effective and well tolerated as a preventive measure for migraine [11]. Hejie Zhitong prescription (HJZT) is a Chinese herbal compound that has been used in the clinic for many years, has displayed clear clinical efficacy in the treatment of migraine headaches and has some supporting research results [12–14].Based on the effectiveness of this formulation, the preparation process and quality standard of HJZT (granule preparation) have been developed [15].Experimental studies of pharmacological mechanisms have clarified that HJZT significantly reduces the duration of behavioral changes in nitroglycerin (NTG)-induced model rats and the levels of c-fos and c-jun proteins to exert its analgesic effect [16, 17].

According to published studies, the pathological response of migraine is closely related to the release of related neuropeptide transmitters, such as CGRP [18],substance P(SP) [19],cholecystokinin (CCK) [20] and serotonin (5-hydroxytryptamine,5-HT) [21].Sleep is partially regulated by the brain cytokine network, and cyclooxygenase-2 (COX-2) is involved in the mechanism regulating spontaneous and tumor necrosis factor alpha (TNF-alpha)-induced sleep [22].One researcher also found that sleep deprivation significantly increased the mEPSC frequency and decreased the protein levels of cannabinoid type-1 receptors (CB1Rs) [23].The occurrence of migraine is related to COX-2 [24] and the CB1R [25], which may be the targets of HJZT prescription in migraine treatment. Moreover, stimulation of inflammatory factor production is also an important factor contributing to the progression and recurrence of migraine. This stimulation activates the endogenous pain modulation system. Therefore, in this study, we investigated the mechanism of action of HJZT in migraine treatment from the perspective of nociceptive transmission-associated neurotransmitter inhibition based on the trigeminal neurovascular theory. Rizatriptan monobenzoate tablets (RMTs) contain a 5-HT receptor 1B/D agonist that contracts cerebral blood vessels that expand during migraine attacks [26],which significantly reduces the levels of CGRP and CCK to enhance the inhibition of pain signals via the endogenous pain modulatory system [27].These effects are consistent with the expected outcome of this experiment; thus, RMTs were selected as the positive control drug.NTG has been widely used to induce experimental migraine in animal models [28].Therefore, this study also used NTG to establish animal models, to observe the effect of HJZT on the expression of the aforementioned factors in the TG and midbrain tissues of rats with migraine, and to explore the regulatory mechanism of HJZT.

## Materials and methods

### Main laboratory equipment and drug management

An ELX800 microplate reader was purchased from Bio-TEK, a LightCycler PCR instrument was purchased from Roche Life Technology, and an Olympus BX63 automated fluorescence microscope was purchased from Olympus Japan. HJZT granules (10 g, batch number 20151203) were provided by the Changchun University of Traditional Chinese Medicine Plant Chemistry Research Center, and the HJZT prescription was provided by the Granule Pharmacy of the Affiliated Hospital of Changchun University of Traditional Chinese Medicine. Wuweizi granules were obtained from Gansu Xifeng Pharmaceutical Co., Ltd.(batch number 20150819).Pentobarbital sodium was obtained from Sigma (batch number WXBB6772V). RMTs were obtained from Hubeidianli Pharmaceutical Co., Ltd.(batch number 170301). NTG was obtained from Beijing Yimin Pharmaceutical Co., Ltd.(batch number 20170216).

### Animals and experimental groups

Sixty mice (male and female) weighing 20 ± 2 g were purchased from Changchun Yisi Laboratory Animal Technology Co., Ltd.[Certificate No. SCXK-(JI)2011-0004; Jilin, China].Mice were randomly divided into 5 groups according to body weight: the blank control group (A), Wuweizi granule (positive control drug, 9.36 g/kg) group (B),high-dose HJZT (9.36 g/kg) group (C),intermediate-dose HJZT (4.68 g/kg) group (D),and low-dose HJZT (2.34 g/kg) group (E).Each group contained 12 mice. Forty-eight rats (male and female) weighing 200–266 g were purchased from Hongda Animal Farm in Kuancheng District, Changchun City [SCXK(JI)2017-0003]. Rats were randomly divided into the blank control group (A),model group (B), RMT (positive control drug,0.1 mg/ml) group (C),high-dose HJZT (2.55 g/ml) group (D),intermediate-dose HJZT (1.28 g/ml) group (E),and low-dose HJZT (0.64 g/ml) group (F),with 8 rats per group. These animal experiments were approved by the Ethics Committee of Changchun University of Traditional Chinese Medicine.

### Animal models and behavioral assays

Mice were intragastrically administered suspensions of HJZT and Wuweizi particles once per day, and the controls received ultrapure water (0.2 ml/10 g body weight) continuously for 1 week. The mice were intraperitoneally injected with pentobarbital sodium (32 mg/kg, a dose below the maximum threshold, according to the results of our preliminary experiment), Wuweizi and different concentrations of HJZT or ultrapure water. The number of animals that fell asleep within 15 min (animals with a positive reflex that disappeared for more than 1 min) was recorded for a coordinated sleep measurement. In addition, the sleep latency and sleep time of mice treated with a concentration above the threshold dose of sodium pentobarbital (44 mg/kg, according to the results of our preliminary experiment) were calculated. Rats in the treatment groups were intragastrically administered 2 ml of different concentrations of HJZT and RMT once per day (1 ml/100 g body weight) for 2 weeks, and the control and model groups were administered the same amount of normal saline. Fourteen days later, rats in the drug intervention and model groups were subcutaneously injected with NTG (10 mg/kg), and rats in the control group were injected with the same amount of normal saline. Then, rats were placed under continuous observation for 30 min. Signs of a behavioral disorder, including frequent scratching of the head with the forelimb, crawling upward, running back and forth, and biting the tail, were recorded. Each behavioral incident received a score of 1 point. The total behavioral scores of each group were calculated.

### Ingredient testing

Small molecule compounds in medicines were identified using high-performance liquid chromatography with tandem mass spectrometry (HPLC–MS/MS).The main ingredients of the prescriptions were qualitatively analyzed, and combined annotation and classification of data were performed with mass spectrometry databases.(This analysis was entrusted to China Qingdao Kechuang Quality Inspection Co., Ltd.)

### ELISAs of CGRP, SP, 5-HT and CCK levels

Experimental rats received continuous treatment with the intervention for 14 days and were subcutaneously injected with NTG 2 h after modeling. Rats in each group were anesthetized with an intraperitoneal injection of 10% chloral hydrate at a dose of 4 mg/kg body weight. Rats were restrained after anesthesia, and the chest cavity was cut, the heart was completely exposed, the right atrial appendage was located, and 4 ml of whole blood were removed with a 5-ml syringe. CGRP and 5-HT ELISA kits were purchased from Elabscience, and SP and CCK kits were purchased from Cloud-clone. All kits were used according to the manufacturer’s instructions.

### RNA isolation and real-time quantitative polymerase chain reaction

The experimental rats were decapitated, and the midbrain and TG tissues were separated, removed and fixed with a 4% paraformaldehyde solution for RNA extraction, which was performed in accordance with the LS1040 Eastep^®^ Super Total RNA Extraction Kit technical manual. Two hundred fifty nanograms of total RNA from each specimen were used as the template for reverse transcription, and 5 × Trans Script All-in-One Super Mix was used to synthesize cDNAs with a qPCR kit. The CCK and CGRP gene-specific primer sequences are listed below.CGRP-F:5′-TCCTGGTTGTCAGCATCTTG-3′,CGRP-R:5′-CTCAGCCTCCTGTTCCTCCT-3′,CCK-F: 5′-AGCTGACTCCGCATCCGAAG-3′,CCK-R:5′-TCATTCCGCCTCCTCCAAGC-3′. For PCR, the reaction system was configured according to the instructions provided with the Roche PCR kit. The cDNA templates (2 μl),the upstream and downstream primers [1 µl (10 µM) each], and FastStart Essential DNA Green Master (10 µl) were mixed and centrifuged, and the reaction system was then placed in a Roche Light Cycler PCR instrument for PCR amplification. The following amplification program was used: denaturation at 95 °C for 10 min; 40 cycles of extension and fluorescence measurement at 95 °C for 10 s, 55 °C for 10 s, and 72 °C for 15 s; extension at 72 °C for 10 min; and a melting curve analysis of the PCR product. Using GAPDH as the internal reference, the target gene mRNA expression level was calculated with the following formula: Fold change = 2^−△△Ct^.

### Immunohistochemical analysis of CB1R and COX-2 expression in midbrain and TG tissues

Tissue samples were routinely dehydrated, embedded in paraffin and sectioned. The sections were dewaxed, rehydrated with a gradient of ethanol solutions, rinsed with water, and subjected to antigen retrieval in citrate buffer. The sections were incubated with anti-CB1 receptor and anti-COX-2 antibodies (1:100; Beijing Biosynthesis Biotechnology Co., Ltd., China) at 4 °C overnight. Then, the biotin-labeled secondary antibody was added in a dropwise manner. After an incubation at room temperature for 10 min, the sections were washed, and staining was visualized with DAB. Finally, the slides were restained with hematoxylin, and the percentage of positively stained cells and the staining intensity in the different groups were determined under a microscope at 400 × magnification.

### HE staining of midbrain and TG tissues

Samples were acquired and prepared as described above. After fixation with 4% paraformaldehyde at 4 °C overnight, the sections were dehydrated, embedded in paraffin using the conventional method, and sliced. Then, the sections were dewaxed, cleared with xylene, rehydrated with ethanol, rinsed with water, stained, dehydrated, observed under a light microscope and imaged.

### Statistical analysis

All analyses and graphical visualizations were performed using GraphPad Prism software version 8.2.1. The significance level was set to 0.05 (two-tailed P value).The data are presented as the mean ± standard deviation (SD) values. The overall effects of different treatments were analyzed using one-way analysis of variance (ANOVA), and the differences between each group were analyzed using Tukey’s post hoc test for multiple comparisons.

## Results

### TIC diagram

**Fig. 1:**
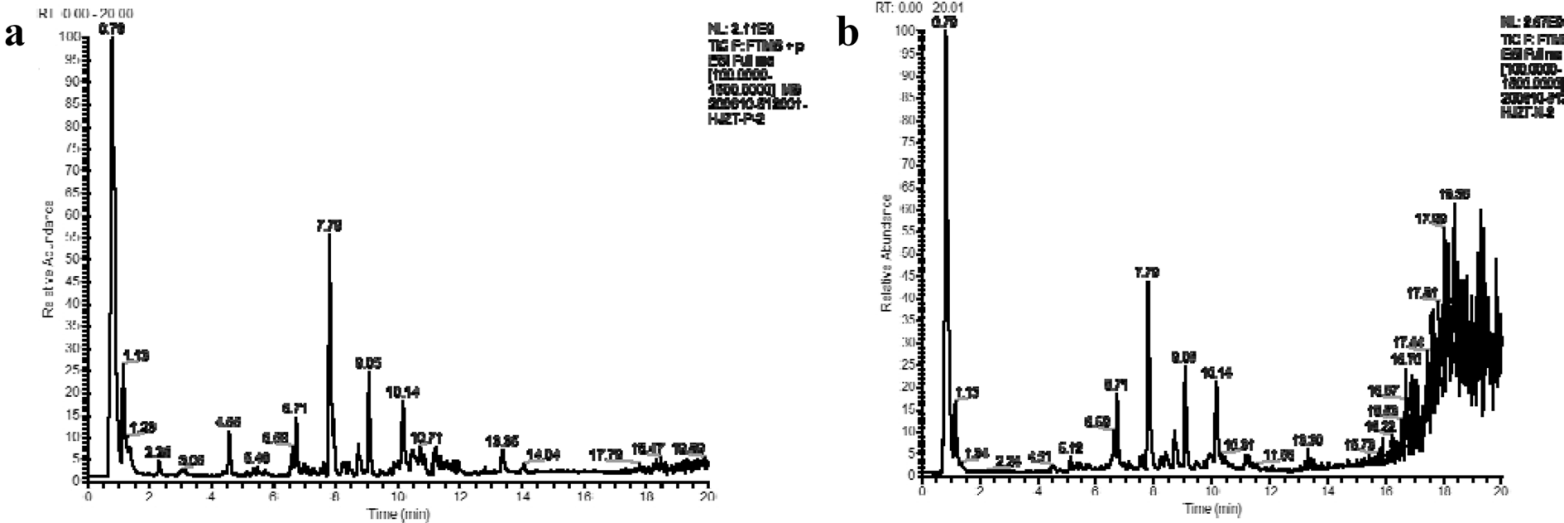
**a** Positive ion mode, **b** negative ion mode

Figure 1a, b show the total ion current (TIC) diagrams for positive and negative ion mode respectively. The main peak were more obvious in positive ion mode, which mainly included flavonoids, carboxylic acids and their derivatives and organooxygen compounds. Specific compositions and classifications are provided in the table presenting the results of the analysis of full spectra (Additional file 1). The six most abundant chemical components (in positive ion mode) are listed in Table 1.

**Table 1:** Relative concentrations of the six most abundant components in HJZT (positive ion mode)

Name	Formula	Molecular weight (actual value)	RT (min)	Molecular weight (theoretical value)	△ppm	Area	Relative concentration
Baicalin	C_21_H_18_O_11_	446.08427	7.816	446.08491	−1	4953871936	13.431
DL-Arginine	C_6_H_14_N_4_O_2_	174.11161	0.75	174.11168	0	2528736051	6.856
D-(+)-Proline	C_5_H_9_NO_2_	115.06352	0.828	115.06333	1	1905536220	5.166
Oroxindin	C_22_H_20_O_11_	460.09985	9.069	460.10056	−1	1840258302	4.989
Galactinol	C_12_H_22_O_11_	342.11568	0.784	342.11621	0	1741454204	4.721
Choline	C_5_H_13_NO	103.10003	0.777	103.09971	3	1038832326	2.816

### HJZT promotes sleep

After the injection of pentobarbital sodium, only eye closing, head bowing, prone positioning and spontaneous activity were inhibited in group A. No reflexes disappeared, and all animals exhibited normal behaviors 30 min after the injection. The behaviors of eye closing, head bowing, prone positioning and spontaneous activity were suppressed. Righting reflexes disappeared in 6 mice, 7 mice, 6 mice and 5 mice in groups B, C, D and E respectively. The drug treatment groups exhibited an increased incidence of sleep compared with the control group (P < 0.01 or P < 0.05). HJZT exerted a synergistic effect with pentobarbital sodium, and the high dose was more effective than the low or intermediate dose (see Table 2).

**Table 2:** Effect of HJZT on the incidence of sleep in mice treated with a subthreshold dose of pentobarbital sodium

Group	Dose (g/kg)	Number of animals	Number of sleeping animals	Incidence of sleep (%)
Control (A)	–	12	0	0
Wuweizi granules (B)	9.36	12	6*	50.00
High-dose HJZT (C)	9.36	12	7**	58.33
Intermediate-dose HJZT (D)	4.68	12	6*	50.00
Low-dose HJZT (E)	2.34	12	5*	41.67

**P < 0.01 compared with the control group and *P < 0.05 compared with the control group

**Fig. 2:**
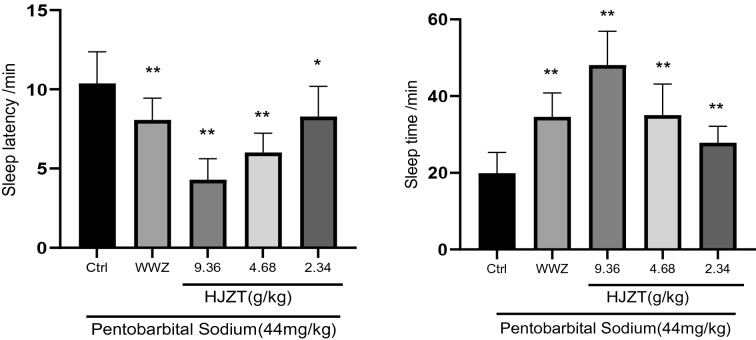
Sleep latency and sleep time of the mice in each group. **P < 0.01 compared with the control group. *P < 0.05 compared with the control group

The drug treatment significantly prolonged the sleep time and reduced the sleep latency of mice treated with a greater than threshold dose of pentobarbital sodium and significantly accelerated the onset of sleep in mice (compared with group A, P < 0.01 or P < 0.05).Based on these results, HJZT and Wuweizi particles exert a synergistic hypnotic effect with pentobarbital sodium, which exerts a good sedative and hypnotic effect alone. See Fig. 2.

### The administration of HJZT attenuates NTG-induced behavioral disorders in rats

After the injection of NTG, rats in group B showed signs of discomfort, such as frequent head scratching with the forelimb, increased cage climbing, and increased back-and-forth movement (no obvious tail biting was observed), while rats in group A were relatively peaceful after the injection of normal saline, and the aforementioned behaviors appeared less frequently. Rats in groups C, D, E and F exhibited more of these behaviors than rats in group A and fewer behaviors than rats in group B. The specific results are listed in Table 3.

**Table 3:** Behavioral disorders observed in the rats in each group

Group	Number of rats	Cage climbing	Head scratching	Running	Tail biting	Behavioral change (total points)
Control (A)	8	37	0	42	0	9.88 ± 2.23
Model (B)	8	148	59	101	0	39.50 ± 4.47^##^
RMT (C)	8	61	2	31	0	11.75 ± 5.55**
HJZT high-dose (D)	8	56	14	40	0	13.75 ± 4.10**
HJZT intermediate-dose (E)	8	46	6	45	0	12.13 ± 1.46**
HJZT low-dose (F)	8	25	27	68	0	15.00 ± 4.90 ^#,^**

^#^P < 0.05 compared with the control group, ^##^P < 0.01 compared with the control group, and **P < 0.01 compared with the model group

### Plasma levels of 5-HT, CGRP, CCK and SP

**Fig. 3:**
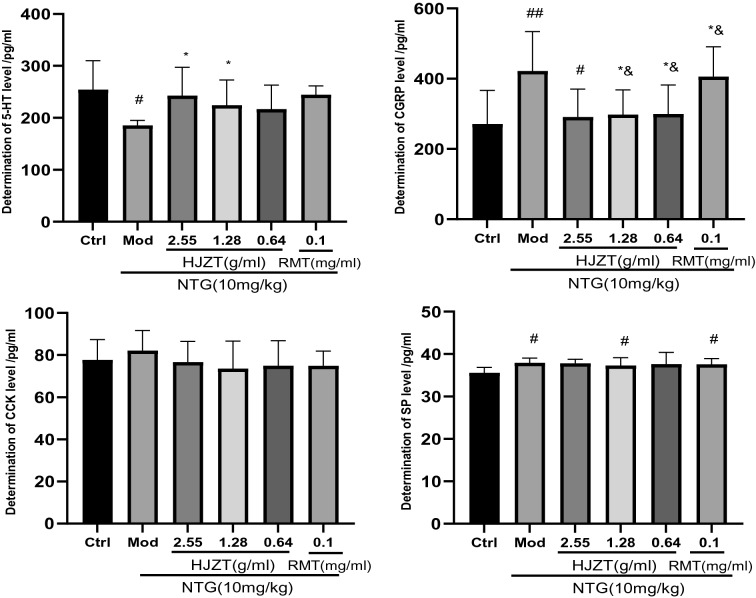
^#^P < 0.05 compared with the control group, ^##^P < 0.01 compared with the control group, *P < 0.05 compared with the model group, and P < 0.05 compared with the RMT group; n = 8, mean±SD

In this experiment, blood was collected 2 h after the rat model of experimental migraine was established by injecting NTG (acute stage of onset) and was tested with ELISA kits. During a migraine attack, the plasma levels of CGRP and SP were increased, plasma 5-HT levels were decreased, and plasma CCK levels were unchanged. HJZT reduced the CGRP levels (P < 0.05) and increased 5-HT levels (high-dose group, P < 0.05) in peripheral blood, but it did not regulate the plasma levels of the SP and CCK proteins (P > 0.05) (Fig. 3).

### Expression of the CGRP and CCK mRNAs in midbrain and TG tissues

**Fig. 4:**
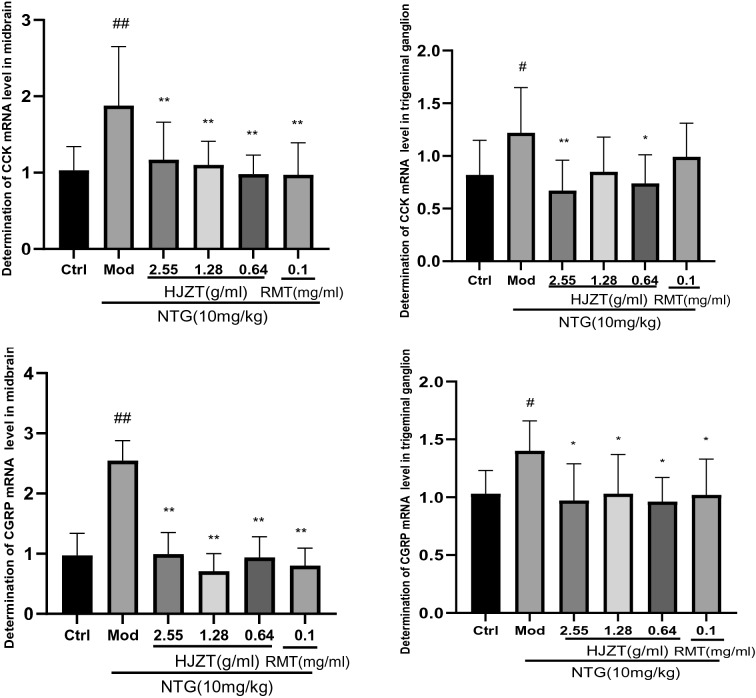
Expression of the CGRP and CCK mRNAs in midbrain and TG tissues. ^##^P < 0.01 compared with the control group, ^#^P < 0.05 compared with the control group, *P < 0.05 compared with the model group, and **P < 0.001 compared with the model group; n = 8, mean±SD

Each HJZT-treated group and the RMT-treated group exhibited reduced levels of the CGRP and CCK mRNAs in the midbrain and TG. In the high-dose HJZT group, the level of the CCK mRNA decreased in the TG, but this decrease was not observed in the RMT group (Fig. 4).

### Expression of CB1R and COX-2 in midbrain and TG tissues

**Fig. 5:**
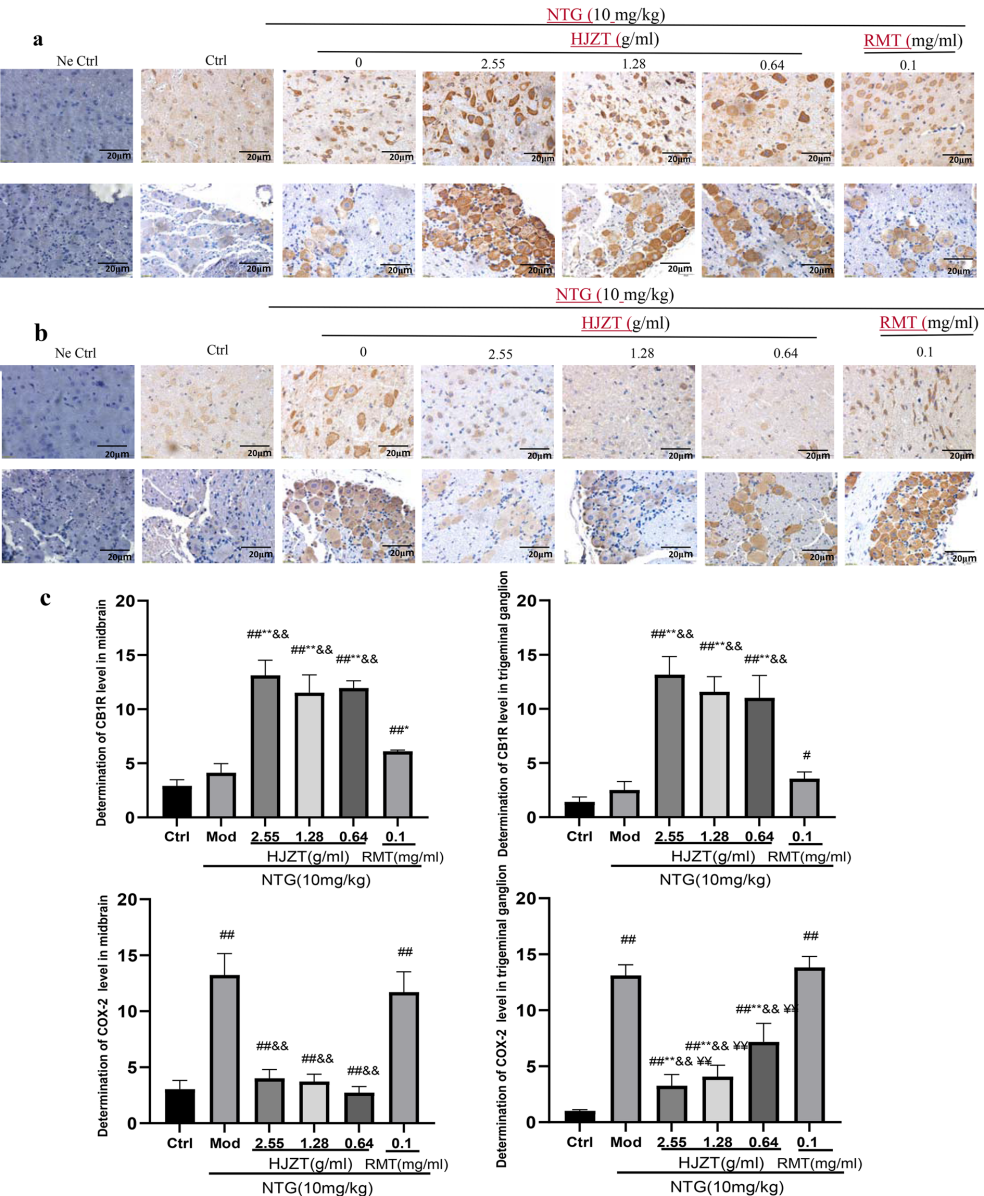
**a** Representative images of CB1R expression (brown staining) in midbrain and TG tissues. **b** Representative images of COX-2 expression in the midbrain and TG. **c** Comparison of the expression of CB1R and COX-2 in the midbrain and TG tissues of rats from each group. ^##^P < 0.01 compared with the control group, ^#^P < 0.05 compared with the control group, *P < 0.05 compared with the model group, **P < 0.001 compared with the model group, ^&&^P< 0.01 compared with the RMT group, and ^¥¥^P< 0.01 compared with the low-dose HJZT group; n = 8, mean±SD

The negative control group consisted of rat midbrain and TG specimens that were not incubated with a primary antibody. Only blue nuclei were observed in this group. Positive expression was primarily detected in the cytoplasm, as evidenced by various shades of brown staining in the cytoplasm. Each dose of HJZT increased the expression of CB1R in the midbrain and TG (grade ++) and reduced the expression of COX-2 in the midbrain (grade −); the high and intermediate doses also inhibited COX-2 expression in the TG. RMT increased the expression of CB1R in the midbrain, but immunohistochemical staining did not reveal positive changes in COX-2 and CB1R expression in the TG (Fig. 5).

### HE staining of midbrain and TG tissues

The HE-stained specimens of the midbrain tissues in each group were observed under a light microscope at a magnification of 400 × .The midbrain neurons in group A were irregularly shaped with synapses, and exhibited a normal morphology, an intact membrane, an abundant, pink-stained cytoplasm and dark blue-stained, round nuclei with full and clear nucleoli. The morphology, number, color, and size of midbrain neurons and nuclei in groups B, C, D, E and F did not differ significantly from neurons in group A. No obvious edema, neuronal apoptosis or necrosis was detected in the brain tissues from any group.

**Fig. 6:**
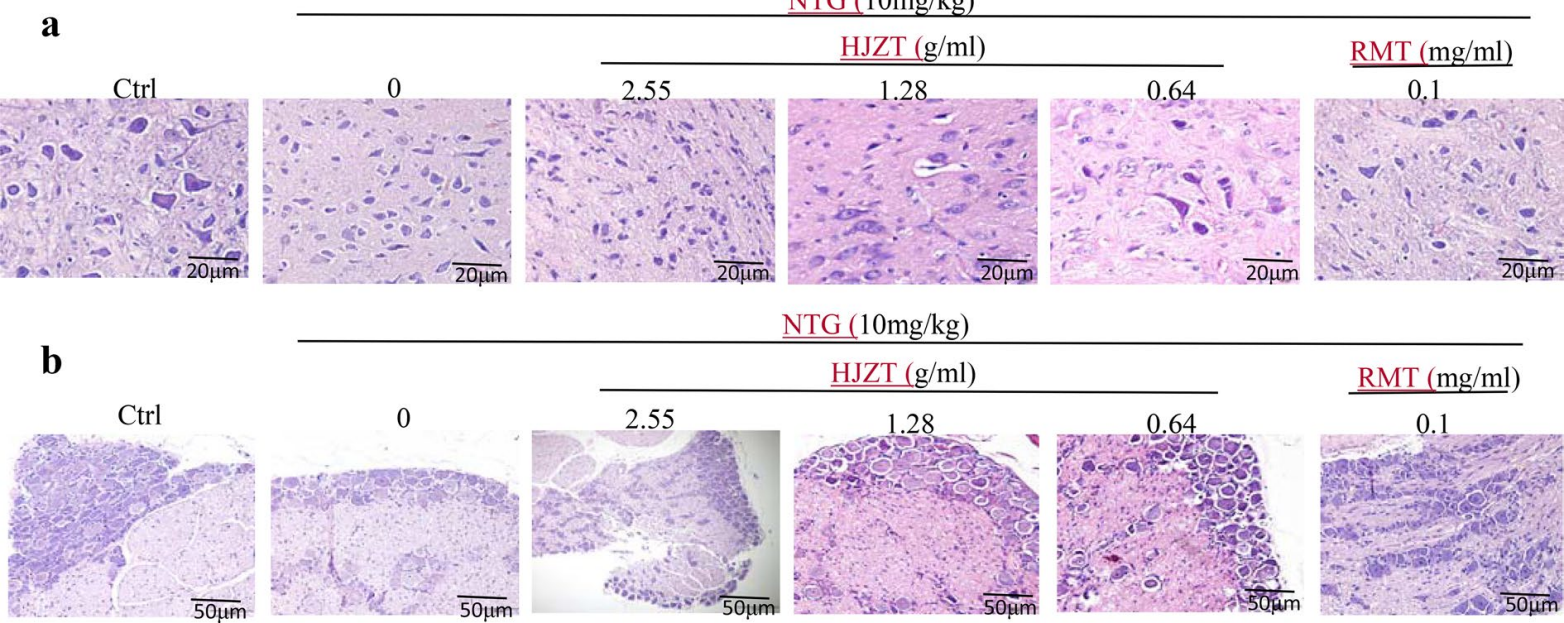
**a** HE staining of midbrain tissues, and B. HE staining of TG tissues

**Fig. 7:**
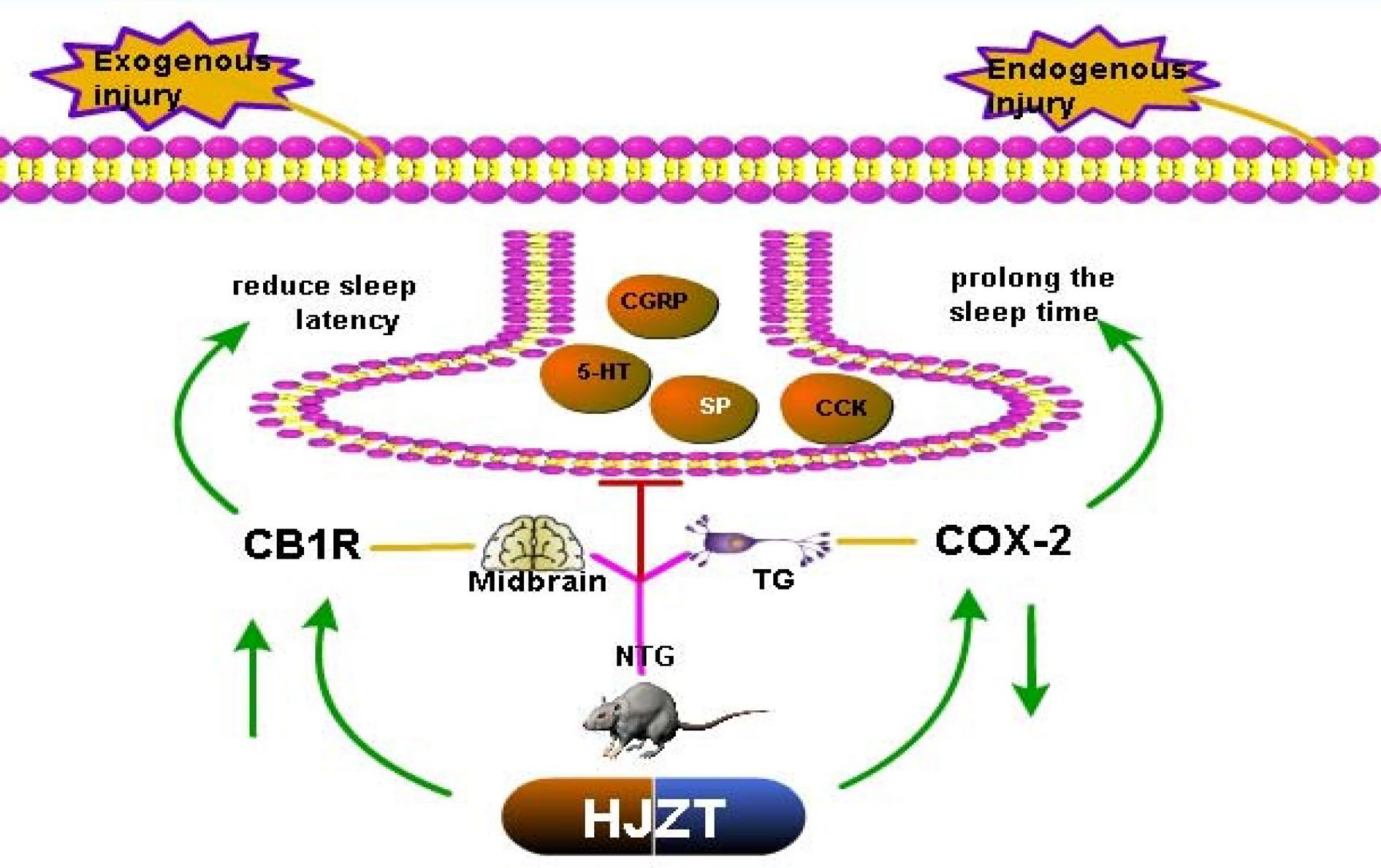
Regulatory mechanism of HJZT in the rat model of NTG-induced migraine

The HE-stained TG specimens from each group were observed under a light microscope at a magnification of 200 × . The bodies of TG cells in group A were round or elliptical, arranged neatly and tightly, and exhibited a normal morphology, an intact membrane, an abundant cytoplasm, and a nucleus. In addition, these tissues exhibited full, clear nucleoli, a pink-stained cytoplasm, dark blue-stained, round nuclei, intact cells that were tightly arranged around the ganglion and relatively sparse in the center of the tissue, and a flat layer of glial cells around the neurons. The shape, number, color, and size of the ganglion cells and nuclei were not significantly altered in groups B, C, D, E and F compared with the cells in group A (Figs. 6 and 7).

## Discussion

HJZT has been used in the clinic for many years. Our team found that it is an effective treatment for patients with migraine. In most patients, it reduces the number of headache attacks and pain. Interestingly, it is able to correct the symptoms of insomnia in patients with migraine, even simple symptoms of insomnia. Therefore, we designed animal experiments using mice. Based on the behavioral changes observed in mice, HJZT effectively promotes the occurrence of sleep in mice, extends the sleep time and reduces the sleep latency. After clarifying its role in sedation and sleep promotion, we further wanted to study the anti-migraine effect of HJZT.

In the process of migraine, trigeminal activation releases several neuropeptides. For example, increased peripheral CGRP levels have been observed during migraine attacks [29], and midbrain structures have been extensively discussed as structures that potentially drive or generate migraine [30]; therefore, an experiment using rats was designed. In the rat model of migraine, HJZT downregulated the expression of the CGRP and CCK mRNAs and upregulated 5-HT. We concluded that HJZT mediates pain resistance by affecting nociceptive neurotransmitters. Our team further verified the mechanism by which HJZT inhibits pain-associated neurotransmitters. COX-2 is an inducible enzyme, and its expression is closely related to the severity of inflammation [31].A study examining COX-2 gene polymorphisms in patients with migraine showed that the COX-2-765 G + genotype was related to an increased risk of migraine [32].In the model of NTG-induced migraine, COX-2 expression was significantly upregulated, but its expression was downregulated by HJZT, and COX-2 plays a role in the midbrain and TG tissues. Dysregulation of endocannabinoid signaling may contribute to the etiology and pathophysiology of migraine [33].Two subtypes of cannabinoid receptors, CB1R and CB2R, have been identified. In addition, in a study of actual clinical cases, rare heterozygous coding variants in CNR1, which encodes CB1R, were significantly associated with pain sensitivity, particularly in patients with migraine [34]. HJZT increases the expression of CB1R in the midbrain and TG.

According to the conclusions obtained from the experiments, HJZT does not regulate the plasma SP and CCK levels. In this experiment, blood was collected 2 h after the successful establishment of the model, because this time point occurs during the onset period of migraine in the experimental model rats. However, a literature review revealed a short half-life of CCK [35], which may explain why plasma CCK levels were not detected. As for SP, perhaps HJZT does not play a role in SP, and perhaps other protein detection methods can be used to explore whether the modified SP protein has changed.

HE staining of midbrain and TG did not reveal obvious histomorphological abnormalities. Based on these observations, the behavioral changes and symptoms of migraine are caused by dysfunction, which further supports the trigeminal neurovascular theory. Data published by Simon Akerman reveal a novel interaction between the serotonergic and endocannabinoid systems in the processing of somatosensory nociceptive information [36].According to the results of our experiments, HJZT reduced COX-2 expression and increased CB1R expression in the midbrain and TG to reduce the activity of nociceptive pain neurotransmitters.

Traditional Chinese medicine (TCM) is an efficacious treatment for migraines when administered in accordance with its theory and clinical practices [37].HJZT is based on the formulation of Xiaochaihu Decoction, which exerts good therapeutic effects on “Shaoyang diseases” derived from “Shang Han Lun”. The sovereign drugs of HJZT are Chaihu (*Bupleurum*) and Chuanxiong (*Ligusticum*);the minister drugs are Huangqin (*Scutellaria*),Dangshen (Codonopsis) and Banxia (*Pinellia*).Modern pharmacological studies of sovereign drugs have reported inhibitory effects of saikosaponin (Chaihu) on various inflammatory processes, and a pretreatment with Ssd inhibits the LPS-induced production of inflammatory factors both in vivo and in vitro [38].According to the results of HPLC–MS/MS from HJZT (Additional file 1: Schedule 1),which mainly contains carboxylic acids and their derivatives, organooxygen compounds and flavonoids etc. We postulate that the detected abundance of baicalin, which is mostly derived from Chaihu and Huangqin,was higher in positive ion mode. According to a previous study, baicalin substantially decreases NO and CGRP levels, increases ET levels, and restores the NO/ET balance in rats with migraine [39].DL-Arginine and D-(+)-proline are mainly derived from Chuanxiong, and they have long been used as treatments for migraine and cardiovascular disease [40].The main active ingredient in *Ligusticum chuanxiong* is ferulic acid (FA),which exerts prolonged effects on migraine symptoms [41]. *Pinellia pedatisecta* is a widely used herb in Chinese medicine. Its proinflammatory toxicity is related to *Pinellia pedatisecta* lectin (PPL), which activates the reactive oxygen species (ROS)/mitogen-activated protein kinase (MAPK)/nuclear factor kappa-B (NF-κB) pathway and NOD-like receptor protein 3 (NLRP3) inflammasome [42].The ingredients in the compounds also include benzodiazepines, which the main effects are sedation, hypnosis, and decreased anxiety [43].This is likely to be the mechanism by which HJZT promotes sleep in mice. The aforementioned Chinese medicine monomers may be the main medicinal components that are useful for the treatment of migraine, but further studies are needed to determine whether they are compatible with each other, whether new ingredients are produced, and the specific effects of the components on the clinical efficacy of the compound.

## Conclusions

Migraine often occurs at the same time as insomnia, and treating insomnia may help relieve the main symptoms of migraine. As shown in the present study, HJZT exerts a synergistic effect with pentobarbital sodium on promoting sleep, consistent with the clinical improvement of insomnia symptoms in patients. During migraine attacks, the release of neurotransmitters is a key process, and strategies inhibiting their active expression may play a role in the treatment of migraine. HJZT inhibits the expression of nociceptive transmission-associated neurotransmitters, including 5-HT, CGRP, and CCK, which may be related to its upregulation of CB1R and downregulation of COX-2.

## Supplementary information

**Additional file 1.** Schedule 1.

## Data Availability

The datasets used and/or analyzed during the current study are available from the corresponding author upon reasonable request.

## Abbreviations

HJZT: Hejie Zhitong prescription
TG: Trigeminal ganglion
CGRP: Calcitonin gene-related peptide
NTG: Nitroglycerin
SP: Substance P
CCK: Cholecystokinin
5-HT: 5-hydroxytryptamine
COX-2: Cyclooxygenase-2
TNF-α: Tumor necrosis factor alpha
mEPSC: Miniature excitatory postsynaptic current
CB1R: Cannabinoid type-1 receptor
RMTs: Rizatriptan monobenzoate tablets
HE staining: Hematoxylin-eosin staining
CNR1: Cannabinoid receptor 1 gene
DAB: Diaminobenzidine
FA: Ferulic acid
PPL: *Pinellia pedatisecta* lectin
ROS: Reactive oxygen species
MAPK: Mitogen-activated protein kinase
NF-κB: Nuclear factor kappa-B
NLRP3: NOD-like receptor protein 3
